# A national study of antibiotic use in Greek pediatric hematology oncology and bone marrow transplant units

**DOI:** 10.1017/ash.2022.43

**Published:** 2022-04-26

**Authors:** Elpis Mantadakis, Ioannis Kopsidas, Susan Coffin, Gabriel Dimitriou, Despoina Gkentzi, Emmanouel Hatzipantelis, Aikaterini Kaisari, Antonis Kattamis, Eleni Kourkouni, Smaragda Papachristidou, Evgenia Papakonstantinou, Sophia Polychronopoulou, Emmanuel Roilides, Nikos Spyridis, Sotirios Tsiodras, Maria N. Tsolia, Grammatiki-Christina Tsopela, Theoklis Zaoutis, Athanasios Tragiannidis

**Affiliations:** 1 Democritus University of Thrace Faculty of Medicine, University General Hospital of Alexandroupolis, Alexandroupolis, Greece; 2 Center for Clinical Epidemiology and Outcomes Research (CLEO), Athens, Greece; 3 Division of Infectious Diseases and Center for Pediatric Clinical Effectiveness, Children’s Hospital of Philadelphia, Philadelphia, Pennsylvania, United States; 4 Patras Medical School, University General Hospital of Patras, Patra, Greece; 5 Children & Adolescent Hematology–Oncology Unit, Second Pediatric Department, School of Medicine, Faculty of Health Sciences, Aristotle University of Thessaloniki, Thessaloniki, Greece; 6 Stem Cell Transplant Unit, Aghia Sophia Children’s Hospital, Athens, Greece; 7 First Department of Pediatrics, National and Kapodistrian University of Athens, Aghia Sophia Children’s Hospital, Athens, Greece; 8 Oncology Department, “P & A Kyriakou” Children’s Hospital, Athens, Greece; 9 Department of Pediatric Oncology, Hipppokration General Hospital, Thessaloniki, Greece; 10 Department of Pediatric Hematology–Oncology (T.A.O.), Aghia Sophia Children’s Hospital, Athens, Greece; 11 Third Department of Pediatrics, Aristotle University of Thessaloniki, Hipppokration General Hospital, Thessaloniki, Greece; 12 Infectious Diseases Unit, Second Department of Pediatrics, National and Kapodistrian University of Athens (NKUA), Athens, Greece; 13 Fourth Department of Internal Medicine, National and Kapodistrian University of Athens, Attikon Hospital, Athens, Greece

## Abstract

**Objective:**

We surveyed antimicrobials used in Greek pediatric hematology–oncology (PHO) and bone marrow transplant (BMT) units before and after an intervention involving education regarding the 2017 clinical practice guidelines (CPG) for the management of febrile neutropenia in children with cancer and hematopoietic stem-cell transplant recipients.

**Design:**

Antibiotic prescribing practices were prospectively recorded between June 2016 and November 2017.

**Intervention:**

In December 2017, baseline data feedback was provided, and CPG education was provided. Prescribing practices were followed for one more year. For antibiotic stewardship, days of therapy, and length of therapy were calculated.

**Setting:**

Five of the 6 PHO units in Greece and the single pediatric BMT unit participated.

**Participants:**

Admitted children in each unit who received the first 15 new antibiotic courses each month.

**Results:**

Administration of ≥4 antibiotics simultaneously and administration of antibiotics with overlapping activity for ≥2 days were significantly more common in PHO units in general hospitals compared to children’s hospitals. Use of at least 1 antifungal was recorded in ∼47% of the patients before and after the intervention. De-escalation and/or discontinuation of antibiotics on day 6 of initial treatment increased significantly from 43% to 53.5% (P = .032). Although the number of patients requiring intensive care support for sepsis did not change, a significant drop was noted in all-cause mortality (P = .008).

**Conclusions:**

We recorded the antibiotic prescribing practices in Greek PHO and BMT units, we achieved improved prescribing with a simple intervention, and we identified areas in need of improvement.

The overuse of antimicrobials, the emergence of antimicrobial resistance worldwide, and increasing healthcare-associated costs have shown the importance of maximizing the application of antibiotic stewardship programs (ASPs), which help to maintain the efficacy of currently existing antibiotics and lead to substantial cost savings.^1,2^ This issue is particularly important for children with cancer, who frequently require antibiotics during periods of febrile neutropenia.^3,4^

In pediatric hematology–oncology (PHO) units, evidence-based use of antibiotics and antibiotic de-escalation strategies have the potential to decrease unnecessarily prolonged use of broad-spectrum antibiotics,^5^ but such measures have not been studied extensively.^6^ The few published studies have shown that in hematology–oncology units, antibiotic de-escalation and discontinuation can be safely implemented.^7–10^ For example, a clinical trial in 6 academic hospitals in Spain showed that in adults with hematological malignancies, high-risk febrile neutropenia, and negative blood cultures, empirical antimicrobial therapy can be safely discontinued after 72 hours of apyrexia and clinical recovery irrespective of the absolute neutrophil count (ANC).^11^

In Greece, nearly 300 pediatric oncology patients aged 0–14 years are diagnosed annually. Additionally, ∼12 children with nonmalignant diseases require hematopoietic stem cell transplantation (HSCT) annually. There are 6 PHO units in Greece: 3 units in Athens, 2 units in Thessaloniki, and 1 unit in Heraklion. In addition, there is a single pediatric bone marrow transplant (BMT) unit in Athens. All PHO units in Athens are in children’s hospitals, and the units in Thessaloniki and Heraklion are in general university hospitals.

Since 2016, as part of the project Preventing Hospital Infections in Greece (PHIG), the Center for Clinical Epidemiology and Outcomes Research (CLEO) has been monitoring the use of antimicrobials in Greek PHO units.^12,13^ The goals of this study were to describe the use of antibiotics in hospitalized children with cancer and pediatric HSCT recipients in Greece and to evaluate the impact of a simple multifaceted intervention on prescribing practices.

## Methods

Of the 6 PHO units in Greece, 5 of these units and the country’s single pediatric BMT unit participated in this project. Beginning at the end of June 2016, all participating units prospectively recorded the first 15 children admitted per month who required the initiation of new antibiotics for treatment or prophylaxis. Ethics approval was obtained from the institutional review boards of all participating hospitals.

### Data collection

Children who had already been on intravenous antibiotics the day before the initiation of the new antibiotic regimen were excluded. Administration of any antifungal therapy in a child with febrile neutropenia was recorded as empirical therapy. No pediatric patient was recorded twice within the same month, and the form was completed for the first 7 days of antibiotic administration.

We collected the following data: age and sex, presence of central lines, type of underlying disease (ie, hematologic malignancy, solid tumor, or other disease), ANC and its relation to the first day of antibiotic therapy, antibiotics used and their indication (ie, empirical or targeted therapy, perioperative or other prophylaxis), cultures obtained, pathogens isolated, presence or absence of invasive fungal disease, and clinical outcome (ie, hospital discharge, intensive care unit [ICU] admission, or death).

**Fig. 1.**
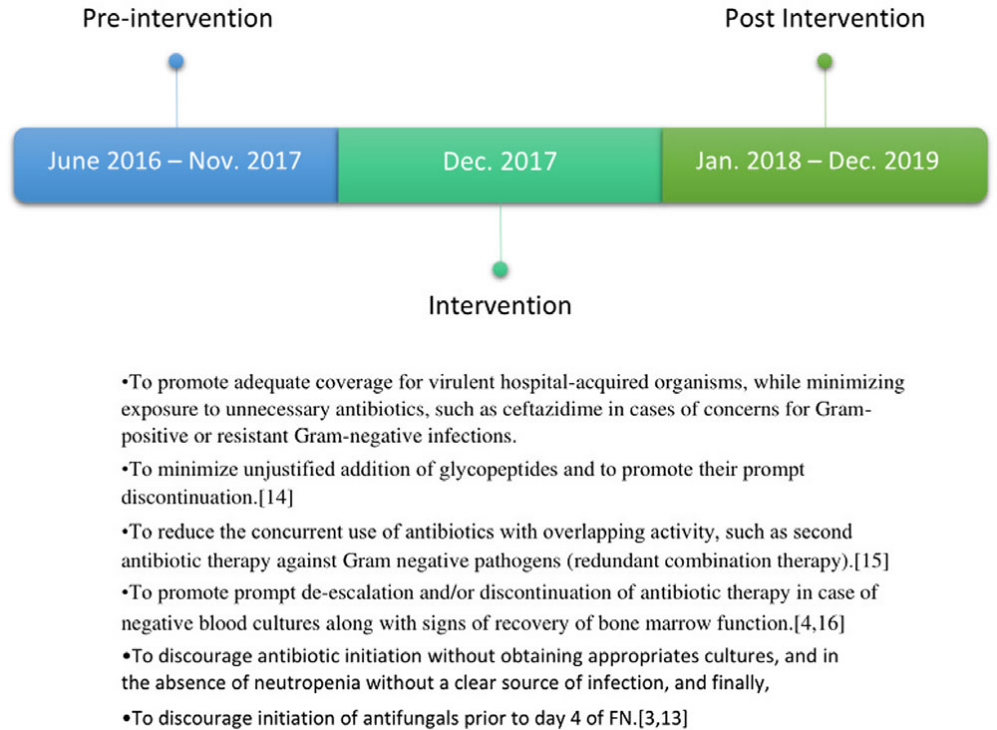
Time frame and goals of the Preventing Hospital Infections in Greece (PHIG) intervention in 5 Greek pediatric hematology–oncology (PHO) units and 1 bone marrow transplant (BMT) unit.

In December 2017, a meeting between CLEO representatives and the directors of all PHO units took place. A baseline data analysis and the main conclusions were presented. Finally, the goal of implementing the International Pediatric Fever and Neutropenia Guideline Panel’s clinical practice guidelines (CPGs) for the management of febrile neutropenia in children with cancer and HSCT recipients, which had recently been updated, was also discussed.^13^ The time frame and the goals of the PHIG intervention are shown in Figure 1.^14–16^ For febrile neutropenia, the definition of the Infectious Diseases Society of America (IDSA) was used.^17^

### PHIG intervention

Surveillance of antibiotic use and the implementation of the CPGs started in December 2017 in 5 of the units. In one unit, surveillance and intervention started in March 2018 due to local resource restrictions; another unit, due to limited resources, participated in data collection only during the pre-intervention period (June 2016 to November 2017). The same demographic and clinical data were recorded in the 2-year postintervention period (January 2018–December 2019) to compare antimicrobial use before and after implementation of the CPGs.^13^ In all participating units, a feedback report was sent every 6 months throughout the study period to inform each unit about its specific progress with respect to the implementation of the CPGs.

### Data analysis

For the purposes of antibiotic stewardship, we calculated days of therapy (DOT) and length of therapy (LOT).^18,19^ One DOT represents the administration of a single antibiotic on a given calendar day, even if multiple doses are given on that day. The number of days a patient is receiving antibiotics, regardless of the number of different agents administered, constitutes LOT. We compared data for the following factors: (1) administration of ≥4 antibiotics simultaneously, (2) use of antibiotics with overlapping antimicrobial activity for ≥2 days, (3) de-escalation or discontinuation with negative cultures by day 6, (4) start of antibiotics without obtaining cultures, (4) antibiotic initiation without a clear source or fever and with ANC > 500/μL, (5) use of standard versus other nonstandard regimens for empirical therapy (defined as cephalosporins without anti-*Pseudomonas* activity, such as ceftriaxone and cefotaxime, ceftazidime monotherapy, colistin, metronidazole, and others), (6) early use of antifungals before day 4 of fever, (7) number of patients admitted for sepsis to ICU, and (8) number of deaths before and after the PHIG intervention.

### Statistical analysis

Nominal variables are presented with absolute and relative (%) frequencies, whereas continuous variables are presented with medians and interquartile ranges. To evaluate differences between units and the effect of the intervention, χ^2^ tests of independence and Mann-Whitney *U* tests were performed, as appropriate. Stratified analysis by unit and type of unit was also performed. All reported *P* values were based on 2-sided tests, and statistical significance was set at *P* < .05. All statistical analyses were performed with Stata version 13.0 software (StataCorp, College Station, TX).

## Results

### Cohort characteristics

Demographic and clinical characteristics of recorded cases by PHO unit and location in a children’s or general hospital are shown in Table 1. Children hospitalized in PHO units in general hospitals were significantly younger than children hospitalized in children’s hospitals: children’s hospitals (median, 7.5 years; IQR, 3.6–12.3) versus general hospitals (median, 5.4 years; IQR, 3.1–8.5) (*P* = .001).

**Table 1.** Demographic and clinical characteristics of cases hospitalized in the six participating PHO units.

	Unit 1		Unit 2		Unit 3 (BMT)		Unit 4		Unit 5		Unit 6		TOTAL	
	N	%	N	%	N	%	N	%	N	%	N	%	N	%
**Gender**														
Male	159	44.8	240	54.2	151	57.4	68	58.6	261	60.1	120	53.1	999	54.4
Female	196	55.2	203	45.8	112	42.6	48	41.4	173	39.9	106	46.9	838	45.6
	**Median**	**IQR**	**Median**	**IQR**	**Median**	**IQR**	**Median**	**IQR**	**Median**	**IQR**	**Median**	**IQR**	**Median**	**IQR**
**Age (years)**	5.2	2.7-8.7	6.4	3.2-11.6	8.1	4-13.2	6.7	4.4-12.7	8.4	3.8-12.6	5.5	3.4-7.8	6.5	3.5-11.7
**Underlying disease**														
Solid tumors	194	54.7	203	45.8	38	14.5	56	48.3	185	42.5	43	19.0	719	39.1
Hematologic malignancies	188	44.5	250	49.0	154	55.9	67	50.9	258	54.5	188	79.2	1105	54.2
Other*	3	0.9	23	5.2	78	29.7	1	0.9	13	3.0	4	1.8	122	6.6
				**Pediatric Hospitals**				**General Hospitals**						
				**N**		**%**		**N**	**%**				***p*** **value**	
**Gender**													0.001	
Male				720		57.3%		279	48%					
Female				536		42.7%		302	52%					
**Underlying disease**													0.001	
Solid tumors				482		38.4%		237	40.8%					
Hematologic malignancies				729		52.5%		376	58%					
Other*				115		9.2%		7	1.2%					

*Other: Aplastic anemia, Fanconi anemia, β-thalassemia, immunodeficiencies (Wiskott Aldrich syndrome, chronic granulomatous disease, severe congenital neutropenia), Langerhans cell histiocytosis, Hemophagocytic lymphohistiocytosis, osteopetrosis, mitochondrial neurogastrointestinal encephalomyopathy.

### Antibiotic use

Patient and antibiotic days, LOT per 1,000 patient days, DOT-to-LOT ratios, and most used antibiotics during the study period by PHO unit and by type of hospital (children’s vs general) are shown in Table 2. Ceftazidime was commonly used in PHO units in general hospitals, whereas piperacillin-tazobactam monotherapy was commonly used in the BMT unit. After the intervention, ceftazidime use decreased from 19% to 1.1% in the 2 PHO units in general hospitals and remained low in units in children’s hospitals throughout the surveillance period (ie, 0.2% before the intervention and 0.4% after intervention). Regarding the use of glycopeptides, their use remained high and essentially unchanged after the intervention. More specifically, glycopeptide use was 30.4% both prior to and after the intervention in PHO units in general hospitals, and this rate increased slightly after the intervention from 24.9% to 27.2% in PHO units in children’s hospitals.

**Table 2.** Use of antibiotics before (PRE) and after (POST) PHIG intervention. Unit 4 did not participate in data collection after intervention.

	Unit 1		Unit 2		Unit 3 (BMT)		Unit 4		Unit 5		Unit 6		TOTAL		Pediatric hospitals		General hospitals	
	Pre	Post	Pre	Post	Pre	Post	Pre	Post	Pre	Post	Pre	Post	Pre	Post	Pre	Post	Pre	Post
Patients	169	186	187	256	103	160	116		173	262	100	126	848	990	579	678	269	312
Antibiotic days	2030	2446	4135	6238	4349	5181	7539		1093	1291	1528	2201	20674	17358	17116	12710	3558	4647
Patient days	4253	5775	6832	11929	4450	5310	18019		2920	5407	3485	6255	39959	34677	32221	22646	7738	12030
LOT/ 1,000 patient days	477	424	605	523	977	976	418		374	239	438	352	517	501	531	561	460	386
DOT/LOT ratio	2.6	2.8	2.1	1.7	1.5	1.5	1.6		2.1	2.2	2.2	1.3	2.0	2.0	1.9	1.9	2.4	2.2
Most commonly used antibiotics - % of DOTS as recorded during first 7 days of treatment																		
Amik/Genta	27.7	28.1	14.6	9.6	6.5	3.5	22.6		26.4	28.8	24.4	19.4	21.7	20.2	18.6	16.6	26.6	25.9
Cefepime	0.1	0.0	0.5	0.6	0.4	0.4	0.0		25.5	20.9	6.9	6.4	6.1	6.5	8.4	9.5	2.4	1.6
Ceftr/Cefotax	7.6	3.6	3.0	8.9	2.5	1.0	9.8		2.8	1.4	3.3	6.4	5.0	4.0	4.2	3.8	6.2	4.3
Ceftazidime	18.9	0.0	0.0	0.0	0.6	1.5	0.4		0.0	0.0	19.0	4.5	7.3	0.6	0.2	0.4	19.0	1.1
Cipro/Levo	1.3	1.5	0.6	0.5	1.5	10.1	0		1.8	0.5	0.9	0.6	1.1	2.1	1.0	2.7	1.2	1.3
Colistin	0.7	2.3	0.3	0.0	1.5	0.5	0.4		0.0	0.0	0.2	0.5	0.4	0.8	0.4	0.1	0.5	1.8
Meropenem	4.7	4.5	5.4	4.4	6.4	7.9	1.3		4.0	3.9	7.8	13.8	4.8	5.7	4.3	5.0	5.7	6.8
Metronidazole	4.2	4.2	4.1	1.3	1.2	4.7	2.3		1.5	1.2	3.2	8.4	3	3.3	2.4	2.1	3.9	5.3
Pip/Tazo	0.7	24.2	33.3	39.8	52.4	39.7	46.6		9.3	15.4	2.4	37.1	20	28.4	31.5	29.1	1.3	27.4
Vanc/Teico	32.8	30	35.9	30.8	23.2	25.6	10.9		23.9	25.3	25.9	31.7	27	28.4	24.9	30.4%	27.2	30.4

**Amik/Genta=**Amikacin/Gentamycin**; Ceft/Cefotax=**Ceftriaxone/Cefotaxime**; Cipro/Levo=** Ciprofloxacin/ Levofloxacin**; Pip/Tazo=** Piperacillin/Tazobactam **Vanc/Teico=**Vancomycin/Teicoplanin.

### Redundant antibiotic use

The results of the PHIG intervention in relation to other study goals are summarized in Table 3. Overall, the simultaneous administration of ≥4 antibiotics remained unchanged at 4.1% before and after the intervention. However, this rate changed from 2.2% to 0.9% in PHO units in children’s hospitals and from 8.2% to 11.2% in units in general hospitals. Both the pre- and postintervention comparisons of the simultaneous use of ≥4 antibiotics between units by type of hospital were highly significant (*P* = .001 for both). The overall administration of antibiotics with overlapping activity for ≥2 days did not change significantly (4.5% before and 5.4% after the intervention; *P* = .39). This rate decreased from 3.5% to 2.1% in units in children’s hospitals but increased from 6.7% to 12.5% in units in general hospitals. Again, the pre- and postintervention comparisons of the administration of antibiotics with overlapping activity for ≥2 days between units by type of hospital were highly significant (*P* = .034 and *P* = .001, respectively). Notably, all study goals improved in unit 2 after the intervention (Table 3).

**Table 3.** Administration of ≥ 4 antibiotics simultaneously, use of antibiotics with overlapping activity for ≥2 days, start of empirical therapy without obtaining cultures and antibiotic initiation without clear source or fever and with ANC>500/μl before and after PHIG intervention.

	Unit 1			Unit 2			Unit 3-BMT			Unit 5			Unit 6		
	PRE	POST	*p*-value	PRE	POST	*p*-value	PRE	POST	*p*-value	PRE	POST	*p*-value	PRE	POST	*p*-value
Administration of ≥ 4 antibiotics simultaneously (for 2 days) (TMP/SMX* not included)	8.9%	17.2%	0.021	5.3%	0.8%	0.003	0.0%	1.3%	0.255	1.8%	0.8%	0.352	7.0%	2.4%	0.094
Antibiotics with overlapping activity for ≥2 days	8.9%	12.4%	0.288	6.4%	1.6%	0.007	1.0%	5.0%	0.079	1.2%	0.8%	0.675	3%	12.7%	0.009
Empirical therapy started without obtaining cultures	36.1%	23.6%	0.014	9.3%	1.2%	0.001	3.1%	7.1%	0.176	10.6%	9.5%	0.706	9.5%	10.5%	0.805
Antibiotic initiation without clear infectious source or fever and with ANC>500/μl	4.9%	3.4%	0.499	7.5%	6.6%	0.744	0%	0%	–	0%	1.2%	0.167	5.3	2.4	0.266

*TMP/SMX: Trimethoprim/sulfamethoxazole (cotrimoxazole).

### Antibiotic use for febrile neutropenia

The antibiotic use rates on day 1 of febrile neutropenia before and after the PHIG intervention are shown in Supplementary Table S1. Major differences in practice existed between different units prior to intervention. In units 1 and 2, triple antibiotic therapy (ie, an antipseudomonal β-lactam, a second gram-negative agent, and a gram-positive agent) was commonly practiced. In the BMT unit, monotherapy with an antipseudomonal β-lactam was more commonly used. In unit 5, double gram-negative coverage was used, and in unit 6, triple antibiotic therapy and other nonstandard regimens were frequently used. Significant improvements in antibiotic prescribing were noted after the intervention (*P* = .005). For all units combined, antibiotic therapy was started without obtaining cultures less frequently after the intervention (14.1% before vs 9.8% after; *P* = .006). This practice was more frequent in PHO units in general compared to children’s hospitals before the intervention (25.5% vs 9%; *P = .*001) and after the intervention (18.2% vs 5.8%; *P* = .001).

### De-escalation of antibiotic therapy

In cases of negative blood cultures in patients receiving antibiotics with gram-positive coverage and/or a second antibiotic with gram-negative coverage, de-escalation and/or discontinuation of antibiotics on day 6 in all units significantly increased from 43% to 53.5% (*P* = .032). This rate of discontinuation increased from 53.1% to 60.2% in units in children’s hospitals and from 15.1% to 28.3% in units in general hospitals.

Antibiotic initiation without a clear infectious source or fever and with ANC >500/μL occurred during the preintervention period in 4.1% of antibiotic courses and in the postintervention period in 3% of antibiotic courses (*P* = .198). In PHO units in children’s hospitals, this rate decreased from 3.7% to 3%, and in PHO units in general hospitals, this rate decreased from 5% to 3%.

### Use of nonstandard antibiotic regimens

The overall use of nonstandard antibiotic regimens was not significantly different after the intervention (11.9% before vs 12.7% after). However, the use of nonstandard antibiotic regimens was significantly more frequent in PHO units in general hospitals than in children’s hospitals prior to the intervention (*P* = .001) and remained so after the intervention (*P* = .001). Furthermore, triple antibiotic therapy decreased in units 1 and 2; monotherapy with an antipseudomonal β-lactam decreased in the BMT unit; and triple antibiotic therapy and nonstandard antibiotic combinations decreased in unit 6.

### Antifungal use

The use of antifungals in PHO units before and after the PHIG intervention is shown in Supplementary Table S2. Overall, 47.4% of patients before the intervention and 46.9% after the intervention received at least 1 antifungal medication (*P* = .893). Almost all patients treated in the BMT unit were receiving at least 1 antifungal medication at the time of data collection. Almost 66% of children treated in units in general hospitals were receiving at least 1 antifungal medication, compared with ∼25% of patients treated in units in children’s hospitals. Overall, the use of at least 1 antifungal medication was significantly more common in PHO units in general versus children’s hospitals before the intervention (66% vs 40.9%; *P* = .001) and remained so after the intervention (73% vs 36.2%; *P* = .001).

### Outcomes (ICU admissions and deaths)

The number of patients requiring ICU support for sepsis did not change significantly: 18 patients (2.13%) before intervention versus 23 patients (2.33%) after the intervention (*P* = .781). We noted a significant decrease the number of deaths in all PHO units combined: 36 deaths (4.29%) before the intervention versus 21 deaths (2.13%) after the intervention (*P* = .008).

## Discussion

We prospectively recorded inpatient antibiotic use in pediatric oncology patients and HSCT recipients hospitalized in 5 of the 6 PHO units and in the single BMT unit in Greece. These units treat >92% of children with hematologic and oncologic diseases in the country, so these data can be considered highly representative of the whole country. The distribution of underlying diseases differed significantly by unit, likely because patients with diagnoses other than hematologic malignancies and solid tumors were almost exclusively treated in hospitals in Athens, predominantly in the BMT unit.

The first goal of this study was to document the use of antibiotics in PHO units throughout Greece. The second goal was to educate the medical personnel of all PHO units on the evidence-based management of febrile neutropenia in children with cancer and HSCT recipients, according to the updated international CPG on febrile neutropenia.^13^ For the latter goal, feedback reports were sent regularly to all participants to inform them about their specific progress.

In our study, LOT per 1,000 patient days slightly decreased after the intervention from 517 to 501. During the same period, we noted a highly significant decrease in the number of deaths in all PHO units combined. The number of patients requiring ICU support for sepsis did not change significantly despite the overall decreased use of antibiotics. The fact that ICU admissions did not increase despite the use of fewer antibiotics is a strong indication that our intervention was safe. However, improved care and increased expertise resulting from the implementation of oncology protocols are other possible reasons for the drop in overall mortality.

The use of ceftazidime decreased after the intervention. Notably, most Greek hospitals harbor multidrug-resistant *Pseudomonas aeruginosa* with decreased susceptibility to ceftazidime.^20,21^ The goal of decreasing the use of glycopeptides was not achieved; their use represented 25%–30% of antibiotic DOT before and after the intervention. This failure is indicative of the difficulties frequently encountered in the practical implementation of evidence-based antibiotic guidelines. Established and unproven practices are difficult to modify, especially from physicians without adequate training in antibiotic stewardship.

Based on IDSA and the Society for Healthcare Epidemiology of America guidelines,^22^ these data are insufficient to recommend combination antibiotic therapy as routine to prevent the emergence of resistance, although empirical combination therapy is important for critically ill patients at risk of infection with multidrug-resistant pathogens. In addition, de-escalation and/or discontinuation of empirical antimicrobial therapy based on culture results, and the elimination of redundant combination therapy, are highly recommended and can result in decreased antimicrobial exposure and substantial cost savings.^22^ As a result, a goal of the PHIG intervention was to minimize redundant combination antibiotic therapy. This goal was partially achieved: the administration of ≥4 antibiotics simultaneously and of redundant combination therapy decreased in units in children’s hospitals but increased in units in general hospitals. The reasons for the remarkable improvement in antibiotic use in unit 2 are unclear, although the availability of adequate medical personnel likely played a role.

Overall, the reasons for these inconsistent changes are uncertain. We speculate that the involvement of pediatric infectious disease specialists was higher in units in children’s hospitals. Another possible explanation is that on-call physicians treating patients with febrile neutropenia in general hospitals are more likely to choose more aggressive antibiotic therapies than those needed for lower-risk patients with febrile neutropenia. The latter was shown to be the case in the management of adults with febrile neutropenia in an urban tertiary-care teaching hospital in the United States that provides emergency and inpatient services to a large comprehensive cancer center.^23^ It could be argued that unit antibiograms show higher rates of resistance in units situated within general hospitals compared to units in children’s hospitals. However, to our knowledge, no such data exist because PHO units are not typically located within general hospitals outside Greece. Another possible explanation is that general hospitals likely harbor more drug-resistant bacteria, necessitating more aggressive empirical antibiotic therapy, as had been previously shown for uropathogens.^24,25^

Notably, the BMT unit used more piperacillin-tazobactam monotherapy, an evidence-based strategy, compared to any of the PHO units. Although the risk of infections in allogeneic HSCT patients is higher than in PHO patients and their outcomes tend to be worse,^26^ a review of randomized trials showed that monotherapy for high-risk febrile neutropenia is an effective strategy.^27^

The prompt de-escalation and/or discontinuation in cases of negative blood cultures increased in all units but remained low after the intervention. Regarding antibiotic initiation without a clear infectious source or fever and with ANC >500/μL, no significant change was noted after intervention, but this practice was relatively rare to begin with.

The use of antifungals on days 1–4 of fever is contrary to international guidelines,^13,28,29^ but almost 66% of children treated in units in general hospitals received at least one antifungal compared to ∼25% of patients treated in units in pediatric hospitals. This practice did not change significantly after the intervention. Such early use of antifungals (ie, prior to day 4 of febrile neutropenia) is unjustified. Apart from children with acute myeloid leukemia, refractory, or relapsed acute lymphoblastic leukemia, Burkitt lymphoma, and exposure to high-dose corticosteroids,^30–32^ most children with cancer do not require prophylaxis or therapy with antifungal agents. A multicenter randomized controlled trial in Italy showed that empirical antifungal therapy was of no advantage in terms of survival without fever and invasive fungal disease in children who were defined as low risk for systemic fungal disease.^33^

Our study had several strengths and limitations. This was the first study to prospectively collect data regarding antibiotic use in Greek PHO units. Although one PHO unit did not participate and another did not collect data during the postintervention period, our data are representative of the inpatient antibiotic prescribing practices for children with cancer and HSCT recipients treated in specialized units in Greece. One limitation of the study is that only hospital-wide and not unit-specific antibiotic susceptibility data for bacterial pathogens were available. Hence, we could only speculate about the reasons for the considerably different antibiotic prescribing practices among units. Lower susceptibility rates of microbial pathogens may be the reason for the more aggressive use of antibiotics in general hospitals with PHO units, but this issue needs further research. Finally, we were unable to separate all-cause from infectious mortality.

In conclusion, we used surveillance data to describe prescribing practices in Greek PHO and BMT units. We implemented a simple intervention that was associated with improved prescribing practices. We also identified areas necessitating improvement. As a next step, an active intervention with designated antibiotic stewardship physicians and prospective audit-and-feedback recommendations at each Greek PHO unit is needed.

## Supplementary material

For supplementary material accompanying this paper visit https://doi.org/10.1017/ash.2022.43. click here to view supplementary material
