# Supplementary material for: A national study of antibiotic use in Greek pediatric hematology oncology and bone marrow transplant units
Source: Antimicrob Steward Healthc Epidemiol. 2022 Apr 26;2(1):e71. doi: 10.1017/ash.2022.43 (PMC9726537; doi:10.1017/ash.2022.43)
Supplement: Supplementary file 1 [file ashsup.zip › S2732494X22000432sup001.docx]

529 **TABLE 5** Use of antifungals in Greek PHO units before (PRE) and after (POST) PHIG intervention.

|  | **Unit 1** | | **Unit 2** | | **Unit 3 (BMT)** | | **Unit 5** | | **Unit 6** | | **Total** | |
| --- | --- | --- | --- | --- | --- | --- | --- | --- | --- | --- | --- | --- |
|  |  |  |  |  |  |  |  |  |  |  |  |  |
|  | PRE | POST | PRE | POST | PRE | POST | PRE | POST | PRE | POST | PRE | POST |
|  |  |  |  |  |  |  |  |  |  |  |  |  |
| **Amphotericin B** | 0.0% | 1.9% | 6.7% | 1.3% | 7.1% | 3.0% | 0.9% | 1.2% | 3.2% | 6.2% | 4.0% | 3.7% |
|  |  |  |  |  |  |  |  |  |  |  |  |  |
| **Fluconazole** | 9.5% | 9.6% | 3.3% | 9.1% | 78.6% | 73.1% | 10.3% | 12.8% | 2.1% | 4.1% | 16.7% | 18.9% |
|  |  |  |  |  |  |  |  |  |  |  |  |  |
| **Caspofungin** | 0.0% | 0.0% | 0.0% | 0.0% | 0.0% | 0.0% | 0.0% | 0.0% | 0.4% | 11.3% | 3.2% | 1.4% |
|  |  |  |  |  |  |  |  |  |  |  |  |  |
| **Micafungin** | 0.0% | 17.3% | 15% | 7.8% | 12.5% | 10.4% | 1.9% | 3.7% | 14.9% | 23.7% | 17.2% | 17.8% |
|  |  |  |  |  |  |  |  |  |  |  |  |  |
| **Voriconazole** | 57.1% | 34.6% | 1.7% | 3.9% | 3.6% | 11.9% | 0.0% | 0.0% | 2.5% | 29.9% | 9.5% | 6.7% |
|  |  |  |  |  |  |  |  |  |  |  |  |  |
| **Posaconazole** | 0.0% | 0.0% | 0.0% | 0.0% | 1.8% | 3% | 0.9% | 0.0% | 0.7% | 0.0% | 0.5% | 0.5% |
|  |  |  |  |  |  |  |  |  |  |  |  |  |
| **At least one antifungal** | 66.7% | 61.5% | 26.7% | 22.1% | 98.2% | 98.5% | 14% | 17.1% | 64.3% | 80% | 47.4% | 46.9% |
|  |  |  |  |  |  |  |  |  |  |  |  |  |
| *p* value | 0.607 | | 0.533 | | 0.275 | | 0.501 | | 0.044 | | 0.893 | |
|  |  |  |  |  |  |  |  |  |  |  |  |  |

530

32
