# Supplementary material for: A national study of antibiotic use in Greek pediatric hematology oncology and bone marrow transplant units
Source: Antimicrob Steward Healthc Epidemiol. 2022 Apr 26;2(1):e71. doi: 10.1017/ash.2022.43 (PMC9726537; doi:10.1017/ash.2022.43)
Supplement: Supplementary file 1 [file ashsup.zip › S2732494X22000432sup002.docx]

527 **TABLE 4** Antibiotic use on day one of FN before (PRE) and after (POST) PHIG intervention.

| **Therapy on day 1 of FN** | **Unit 1** | | **Unit 2** | | **Unit 3** | | **Unit 5** | | **Unit 6** | | **Total** | |
| --- | --- | --- | --- | --- | --- | --- | --- | --- | --- | --- | --- | --- |
|  |  |  |  |  |  |  |  |  |  |  |  |  |
|  | PRE | POST | PRE | POST | PRE | POST | PRE | POST | PRE | POST | PRE | POST |
|  |  |  |  |  |  |  |  |  |  |  |  |  |
| n | 42 | 52 | 60 | 77 | 56 | 66 | 107 | 164 | 55 | 74 | 378 | 433 |
|  |  |  |  |  |  |  |  |  |  |  |  |  |
| **Monotherapy with** | 0.0% | 0.0% | 0.0% | 27.3% | 85.7% | 71.2% | 7.5% | 0.0% | 7.3% | 58.1% | 20.1% | 25.6% |
| **antipseudomonal beta-lactam** |  |  |  |  |  |  |  |  |  |  |  |  |
|  |  |  |  |  |  |  |  |  |  |  |  |  |
| **Antipseudomonal beta-lactam** | 0.0% | 0.0% | 1.7% | 0.0% | 1.8% | 1.5% | 53.3% | 54.9% | 9.1% | 6.8% | 25.9% | 22.2% |
| **and 2nd Gram neg. coverage** |  |  |  |  |  |  |  |  |  |  |  |  |
|  |  |  |  |  |  |  |  |  |  |  |  |  |
| **Antipseudomonal beta-lactam** | 0.0% | 0.0% | 23.3% | 23.4% | 10.7% | 10.6% | 9.3% | 7.9% | 12.7% | 9.5% | 10.3% | 10.4% |
| **and Gram pos. coverage** |  |  |  |  |  |  |  |  |  |  |  |  |
|  |  |  |  |  |  |  |  |  |  |  |  |  |
| **Antipseudomonal beta-lactam** | 83.3% | 67.3% | 66.7% | 36.4% | 1.8% | 3.0% | 21.5% | 32.3% | 34.5% | 10.8% | 31.7% | 29.1% |
| **and 2nd Gram neg. and Gram** |  |  |  |  |  |  |  |  |  |  |  |  |
| **pos. coverage** |  |  |  |  |  |  |  |  |  |  |  |  |
|  |  |  |  |  |  |  |  |  |  |  |  |  |
| **Other combinations** | 16.7% | 32.7% | 8.3% | 13% | 0 | 13.6% | 8.4% | 4.9% | 36.4% | 14.9% | 11.9% | 12.7% |
|  |  |  |  |  |  |  |  |  |  |  |  |  |

528

31
